# Single nucleotide polymorphisms associated with P2X7R function regulate the onset of gouty arthritis

**DOI:** 10.1371/journal.pone.0181685

**Published:** 2017-08-10

**Authors:** Jin-Hui Tao, Miao Cheng, Jiang-Ping Tang, Xiao-Juan Dai, Yong Zhang, Xiang-Pei Li, Qin Liu, Ya-Ling Wang

**Affiliations:** Department of Rheumatology & Immunology, Anhui Provincial Hospital Affiliated to Anhui Medical University, Hefei, Anhui, China; Leibniz-Institut fur Pflanzengenetik und Kulturpflanzenforschung Gatersleben, GERMANY

## Abstract

**Background:**

Gout is an inflammatory disease that is caused by the increased production of Interleukin-1β (IL-1β) stimulated by monosodium urate (MSU) crystals. However, some hyperuricemia patients, even gouty patients with tophi in the joints, never experience gout attack, which indicates that pathogenic pathways other than MSU participate in the secretion of IL-1β in the pathogenesis of acute gouty arthritis. The ATP-P2X7R-IL-1β axis may be one of these pathways.

**Objective:**

This study examines the role of Adenosine triphosphate (ATP) in the pathogenesis of gout and the association of ATP receptor (P2X7R) function with single nucleotide polymorphisms and gout arthritis.

**Methods:**

Non-synonymous single nucleotide polymorphisms (SNP) loci of P2X7R in Chinese people were screened to compare the frequencies of different alleles and genotype distribution of selective SNPs in 117 gouty patients and 95 hyperuricemia patients. Peripheral white blood cells were purified from the peripheral blood of 43 randomly selected gout patients and 36 hyperuricemia patients from the total group. Cells were cultured with MSU or MSU + ATP, and supernatants were collected for the detection of IL-1β concentrations using enzyme-linked immunosorbent assay (ELISA).

**Results:**

1. Eight SNP loci, including rs1653624, rs10160951, rs1718119, rs7958316, rs16950860, rs208294, rs17525809 and rs2230912, were screened and detected, and rs1653624, rs7958316 and rs17525809 were associated with gout arthritis. 2. IL-1β concentrations in supernatants after MSU + ATP stimulation were significantly higher in gouty patients than in the hyperuricemia group [(131.08 ± 176.11) pg/ml vs. (50.84 ± 86.10) pg/ml]; Patients (including gout and hyperuricemia) carrying the susceptibility genotype AA or AT of rs1653624 exhibited significantly higher concentrations of IL-1β than patients carrying the non-susceptibility genotype TT [(104.20 ± 164.25) pg/ml vs. (21.90 ± 12.14) pg/ml]; However, no differences were found with MSU stimulation alone.

**Conclusions:**

ATP promotes the pathogenesis of gouty arthritis via increasing the secretion of IL-1 β, and its receptor (P2X7R) function associated single nucleotide polymorphisms may be related to gouty arthritis, which indicates that ATP-P2X7R signaling pathway plays a significant regulatory role in the pathogenesis of gout.

## Introduction

Gout is an inflammatory disease that is characterized by hyperuricemia and a recurrence of acute gout attacks. Hyperuricemia may lead to the formation and deposition of monosodium urate (MSU) crystals in joints and soft tissues and the consequent clinical manifestations of gout, including episodes of acute gouty arthritis and tophus formation [1–2]. Acute gouty arthritis is a complex inflammatory process, and in vivo and in vitro studies have demonstrated that MSU crystals activate a variety of innate immune cells to release interleukin-1 beta (IL-1β), interleukin-6 (IL-6), interleukin-8 (IL-8), tumor necrosis factor alpha (TNF-α), and other inflammatory mediators [3–4]. IL-1β is the major mediator that induces acute gouty arthritis. The active form of IL-1β is found in joint tissues, including the synovium, synovial fluid, and cartilage, and it is a classical initiator of inflammation [5]. MSU crystals are an endogenous danger signal that is recognized by pattern-recognition receptors (PRRs), including membrane receptors (TLRs) and intracellular receptors (NLRs). The recognition of MSU crystals further activates TLRs and NACHT-LRR-PYD-containing protein 3 (NALP3) inflammasome signaling transduction pathways, which regulate the secretion of IL-1β. The TLR and NLR signaling pathways play critical roles in the development of acute gouty arthritis [6–7].

Some individuals with hyperuricemia develop acute gouty arthritis, but not all individuals with hyperuricemia develop the clinical features of gout [8–10]. Some patients experience no gout attacks during their normal daily schedule despite the presence of MSU crystals in their joints [11–12]. Recent imaging studies revealed MSU crystal deposition and subclinical joint and extra-articular damage in people with asymptomatic hyperuricemia [13–18]. Many people with tophi also never develop acute arthritis [19–21]. These observations indicate that MSU crystals alone are not sufficient to induce acute gouty arthritis. Activation of the NALP3 inflammasome signaling pathway by MSU crystals alone cannot stimulate immune cells to produce sufficient amounts of IL-1β to induce the onset of acute gouty arthritis.

A previous study demonstrated that several signaling pathways regulate IL-1β secretion. MSU and adenosine triphosphate (ATP) stimulate purinergic receptor P2X ligand-gated ion channel 7-induced (P2X7R) IL-1β secretion [22]. The primary predisposing factors for the development of acute gouty arthritis, including strenuous exercise, cold, alcoholism, and overeating, share a common characteristic of the presence of dramatic changes in ATP within the body. This characteristic suggests that changes in ATP may be a second pathogenic signal for acute gouty arthritis. The present study demonstrated that stimulation of peripheral blood leukocytes with MSU alone produced no differences in supernatant IL-1β concentrations between gout and hyperuricemia patients. Gout patients produced higher concentration of IL-1β than hyperuricemia patients following MSU + ATP stimulation. This result suggests that ATP is a second signal in the pathogenesis of gout arthritis after MSU production. Changes in ATP may activate the P2X7 signaling pathway and synergize with MSU crystals to induce the secretion of sufficient IL-1β and further the development of acute gouty arthritis. Therefore, the functional status of P2X7R may determine whether hyperuricemia patients develop acute gouty arthritis, and P2X7R may be a key regulator of acute gouty arthritis [23].

P2X7R is an important member of the P2X family. P2X7R is 595 amino acids in length with two membrane-spanning domains and a longer intracellular C-terminus than the other P2X receptor proteins [24]. The P2X7R gene is highly polymorphic, and many single-nucleotide polymorphisms (SNPs) were detected. P2X7R gene polymorphisms affect the formation of P2X7R membrane pores and K^+^ outflow and alters the functional status of P2X7R [25–26]. For example, mutation of Thr-357 to Ser in the P2X7R amino acid sequence may alter the binding of P2X7R to ATP [27]. Mutation of Glu-496 into Ala impaired ATP-induced release of IL-1β [28–29]. However, mutation of Ala-348 into Thr enhanced P2X7R-stimulated IL-1β secretion [30]. Therefore, the functional status of P2X7R, which is mediated by P2X7R SNPs, may determine whether hyperuricemia patients develop acute gouty arthritis.

The present study classified the common non-synonymous coding SNP loci of gout and hyperuricemia patients and demonstrated that the rs1653624, rs7958316, and rs17525809 SNPs were associated with gout. Patients with these susceptibility loci produced higher concentrations of IL-1β in peripheral blood leukocyte culture supernatants following MSU + ATP stimulation. This result suggests that SNPs associated with P2X7R function regulate the occurrence and development of gout.

## Methods

### Patients and control subjects

This work was approved by the biomedical ethics committee of Anhui Medical University, China. A total of 117 gout patients were recruited from the Department of Rheumatology and Immunology of Anhui Provincial Hospital from September 2015 to December 2016. All gout patients were diagnosed using the 1990 revised American College of Rheumatology classification criteria [10]. All selected cases were male because male individuals are more susceptible to gout than females. Patients with gout aged from 32 to 80 years old, and the mean age was 53.4 ± 15.6 years. Previous studies demonstrated that urate levels were associated with gout and the development of gout within five years [31]. We selected hyperuricemia patients with serum uric acid levels > 540 μmol/L (9 mg/dl) to ensure that these patients would not escalate to gout. The course of disease was longer than 10 years and without a history of gout for the control groups. We recruited 95 hyperuricemia patients from the physical examination center or a related department of the Anhui Provincial Hospital with a mean age of 50.9 ± 14.1 years, aged from 36 to 76 years old. Patients’ clinical data were collected and followed up during the experiment.

### Selection of SNPs investigated

**Fig 1:**
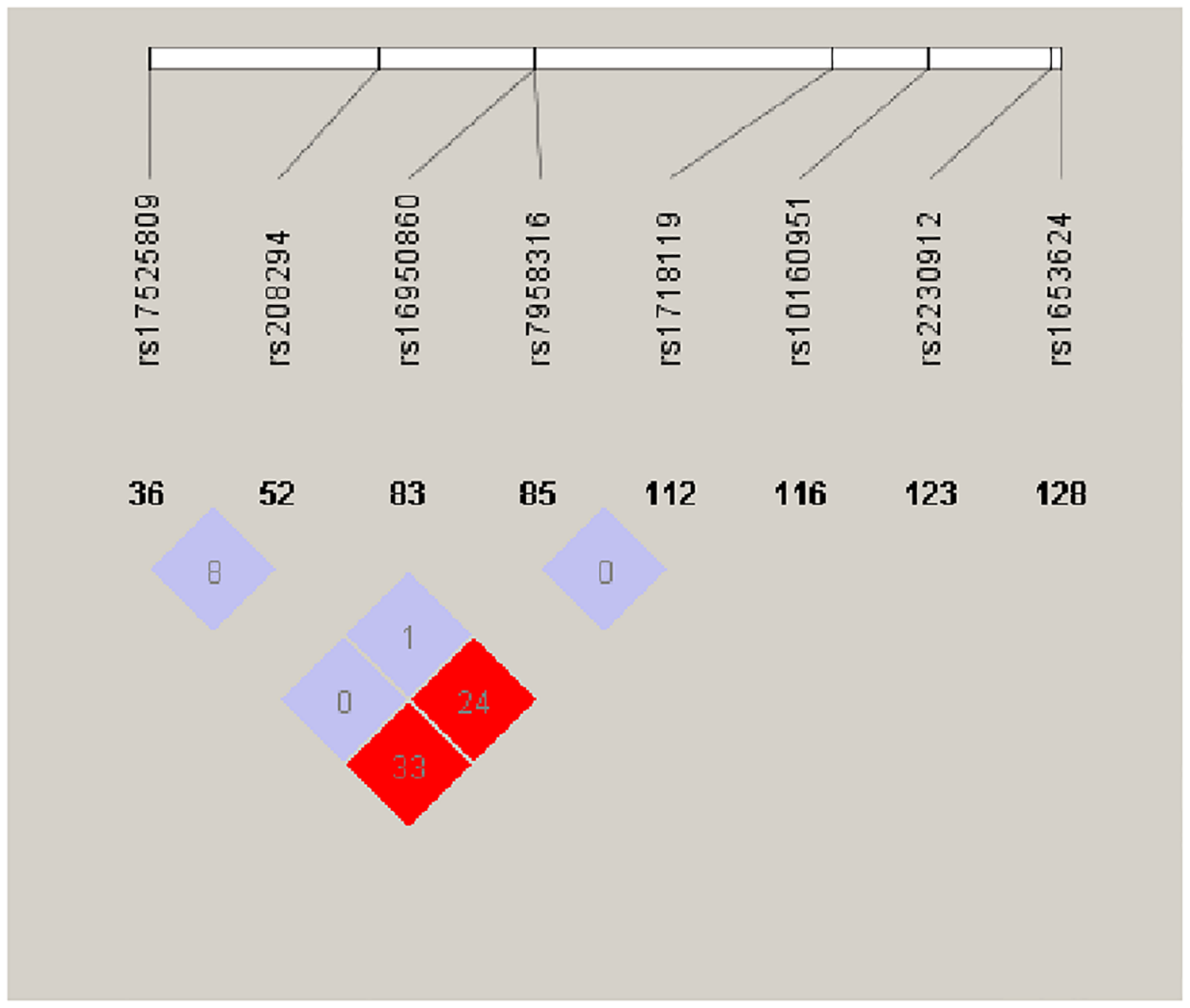
Analysis of the linkage disequilibrium of different SNPs. The results revealed linkage disequilibrium between rs1653624, rs10160951, rs1718119, rs7958316, rs16950860, rs208294, rs17525809, and rs2230912.

P2X7R function is related to changes in its amino acid sequence. Therefore, this study selected non-synonymous coding SNP loci in the P2X7R gene as targets. A search for all SNPs in the P2X7R gene was performed within the Chinese population in the HapMap project database (http://www.hapmap.org/), and the locations of these SNP sites within P2X7R were found in the National Center for Biotechnology Information SNP databank (http://www.ncbi.nlm.nih.gov/SNP/snp_ref.cgi?chooseRs=all&locusId=5027&mrna=NM_002562.5&ctg=NT_029419.13&prot=NP_002553.3&orien=forward&refresh=rerefre). Non-synonymous coding SNP loci were screened out, and eight SNPs were selected: rs1653624, rs10160951, rs1718119, rs7958316, rs16950860, rs208294, rs17525809, and rs2230912. Haploview software was used to analyze the linkage disequilibrium of these loci, and the results revealed linkage disequilibrium (Fig 1).

### DNA extraction and SNP genotyping

Two groups of genomic DNA samples were extracted from peripheral venous blood using a Qiagen DNA Kit (Qiagen, Germany) following the standard DNA isolation instructions. An EP1^™^ high-throughput gene analysis system (Fluidigm, U.S.) was used to genotype genomic DNA using a TaqMan SNP Genotyping Assay Kit (ABI, U.S.). P2X7R gene probes rs1653624, rs10160951, rs1718119, rs7958316, rs16950860, rs208294, rs17525809, and rs2230912 were synthesized for use in this study. Genomic DNA and probe samples were repackaged on 48.48 Dynamic Array^™^ IFC chips and exposed to several cycles in an FC1^™^ PCR Cycler. Genotyping information was collected from the EP1^™^ Reader Data Collection.

### Isolation and stimulation of peripheral blood leukocytes and measurement of cytokine levels

Forty-three gout patients and thirty-six hyperuricemia patients were randomly selected, and hydroxyethyl starch was extracted from peripheral blood leukocytes. The leukocyte concentration was adjusted to 1 × 10^7^/L in cell culture. Cell suspensions were divided into two 500 μl tubes and cultured with uric acid sodium (500 μmol/L) and uric acid sodium (500 μmol/L) + ATP (200 μmol/L) for 24 h. IL-1β concentrations in supernatants were examined using an ELISA (R&D Systems, Inc., Minneapolis, MN, U.S.A.) according to the manufacturer’s instructions.

### Statistical analysis

Comparisons of genotype and allele frequencies between the two groups were performed using chi-square tests in SPSS 10.1 software (SPSS Inc., 2000). Odds ratios (ORs) and 95% confidence interval (CIs) were calculated using non-conditional logistic regression analyses. Hardy—Weinberg equilibrium (HWE) in gout patients and normal controls was determined using SHEsis software (http://analysis.bio-x.cn/myAnalysis.php). Quantitative data are presented as the means ± standard deviation. The t test was used to compare two groups with normally distributed data, and the Mann-Whitney U rank sum test was used to compare groups without normal data distribution. *P* values were calculated based on two-sided tests, and 0.05 was defined as the criterion of significance.

## Results

H-W balance testing for all genotyping results revealed that rs1653624, rs10160951, rs1718119, rs7958316, rs16950860, rs208294, rs17525809, and rs2230912 genotype frequencies were consistent with HWE equilibrium. Only rs10160951 did not conform to the HWE balance, and this gene locus is not discussed further (Table 1).

**Table 1:** Hardy-Weinberg equilibrium test of the genotype frequencies distribution in gout and hyperuricemia patients.

SNPs	GOUT			HWE		Hyperuricemia			HWE	
				χ2	*P*				χ2	*P*
rs1653624	AA	AT	TT			AA	AT	TT		
	47	58	9	2.388	0.122	26	50	16	0.919	0.338
rs10160951	CC	CG	GG			CC	CG	GG		
	113	0	0	NA		93	0	0	NA	
rs1718119	AA	AG	GG			AA	AG	GG		
	3	23	97	1.252	0.263	2	17	74	0.711	0.400
rs7958316	AA	AG	GG			AA	AG	GG		
	25	65	23	2.570	0.108	11	50	33	1.480	0.224
rs16950860	CC	CT	TT			CC	CT	TT		
	86	20	2	0.421	0.516	60	14	2	1.054	0.305
rs208294	CC	CT	TT			CC	CT	TT		
	12	55	45	0.638	0.424	12	37	42	0.699	0.403
rs17525809	CC	CT	TT			CC	CT	TT		
	1	19	89	0.000	0.989	2	37	52	2.473	0.116
rs2230912	AA	AG	GG			AA	AG	GG		
	111	3	0	0.020	0.886	91	1	0	0.003	0.958

### Differences in the distribution of SNP genotypes in P2X7R between gout and hyperuricemia patients

Differences in the prevalence of rs17525809, rs1653624, and rs7958316 were observed between gout and hyperuricemia patients. The gout-sensitivity allele at rs1653624 was A (OR = 1.608, 95%CI: 1.077–2.400). Genotype frequencies were significantly different between gout and hyperuricemia patients. The AA and AT genotypes exhibited a higher risk of gout (AA vs. TT, OR = 3.214, 95%CI: 1.247–8.283; AA + AT vs. TT, OR = 2.456, 95%CI: 1.031–5.853). The gout-sensitivity allele at rs7958316 was A (OR = 1.698, 95%CI: 1.147–2.514), and AA and AG were gout susceptibility genotypes (AA vs. GG, OR = 3.391, 95%CI: 1.402–8.204; AA + AG vs. GG, OR = 2.140, 95%CI: 1.148–3.992; AA vs. AG + GG, OR = 2.229, 95%CI: 1.036–4.796). The gout-sensitivity allele at rs17525809 was T (OR = 2.728, 95%CI: 1.545–4.817), and TT was a gout susceptibility genotype (TT vs. CT + CC, OR = 3.338, 95%CI: 1.763–6.320). There were no significant differences in allele or genotype frequencies between gout and hyperuricemia patients at rs1718119, rs16950860, rs208294, or rs2230912 (Table 2).

**Table 2:** Allele and genotype frequencies and genetic model of SNPs in the P2X7R gene in gout and hyperuricemia patients.

SNPs	Gout	hyperuricemia	χ2 Value	*P* Value	OR	95%CI
rs1653624						
A vs. T	152/76	102/82	5.433	0.02	1.608	1.077–2.400
AA vs. TT	47/9	26/16	6.126	0.013	3.214	1.247–8.283
AT vs. TT	58/9	50/16	2.545	0.111	2.062	0.838–5.072
AA+AT vs. TT	105/9	76/16	4.306	0.038	2.456	1.031–5.853
AA vs. AT+TT	47/67	26/66	3.742	0.053	1.781	0.990–3.204
rs1718119						
G vs. A	197/29	165/21	0.227	0.633	0.865	0.475–1.573
GG vs. AA	97/3	74/2	0.021	0.884	0.874	0.142–5.364
AG vs. AA	23/3	17/2	0.011	0.915	0.902	0.135–6.005
GG+AG vs. AA	120/3	91/2	0.019	0.889	0.879	0.144–5.371
GG vs. AG+AA	97/26	74/19	0.016	0.899	0.958	0.493–1.862
rs7958316						
A vs. G	117/111	72/116	7.043	0.008	1.698	1.147–2.514
AA vs. GG	26/23	11/33	7.620	0.006	3.391	1.402–8.204
AG vs. GG	65/23	50/33	3.599	0.058	1.865	0.976–3.564
AA+AG vs. GG	91/23	61/33	5.838	0.016	2.140	1.148–3.992
AA vs. AG+ GG	26/88	11/83	4.344	0.037	2.229	1.036–4.796
rs16950860						
C vs. T	192/24	146/18	0.002	0.967	0.986	0.516–1.885
CC vs. TT	86/2	66/2	0.069	0.793	1.303	0.179–9.495
CT vs. TT	20/2	14/2	0.114	0.735	1.429	0.179–11.384
CC+ CT vs. TT	106/2	80/2	0.078	0.780	1.325	0.183–9.610
CC vs. CT+TT	86/22	66/16	0.021	0.884	0.948	0.462–1.946
rs208294						
T vs. C	145/79	121/61	0.136	0.712	0.925	0.613–1.397
TT vs. CC	45/12	42/12	0.022	0.881	1.071	0.434–2.646
CT vs. CC	55/12	37/12	0.747	0.388	1.486	0.603–3.664
TT+ CT vs. CC	100/12	79/12	0.294	0.587	1.266	0.540–2.970
TT vs. CT+ CC	45/67	42/49	0.732	0.329	0.784	0.448–1.371
rs17525809						
T vs. C	197/21	141/41	12.592	0.000	2.728	1.545–4.817
TT vs. CC	89/1	52/2	1.112	0.292	3.423	0.303–38.677
CT vs. CC	19/1	37/2	0.000	0.983	1.027	0.087–12.062
TT+ CT vs. CC	108/1	89/2	0.550	0.458	2.427	0.217–27.206
TT vs. CT+ CC	89/20	52/39	14.324	0.000	3.338	1.763–6.320
rs2230912						
A vs. G	225/5	183/1	0.932	0.334	0.246	0.028–2.123
AA vs. GG	111/1	91/0	NA	1.000	0.991	0.974–1.009
AG vs. GG	3/1	1/0	NA	1.000	0.750	0.426–1.321
AA+AG vs. GG	114/1	92/0	NA	1.000	0.991	0.974–1.008
AA vs. AG+ GG	111/4	91/1	1.240	0.265	0.305	0.033–2.776

The genetic patterns of different gout susceptibility gene loci were not identical. The dominant genes rs1653624 and rs7958316 carry dangerous factors. Homozygous and heterozygous hyperuricemia patients with these alleles exhibit a higher risk of gout than patients without these alleles. The rs17525809 locus is a recessive gene, and it is only dangerous for homozygous hyperuricemia patients who exhibit a higher rate of gout. The risk of gout onset may be relatively low in hyperuricemia patients because these patients have a single SNP-susceptible genotype, but a patient with two or more SNP-susceptible genotypes would be more prone to develop gout. Further analysis demonstrated that the more susceptible the genotypes increased gout risk, and the OR reached 5.07 if a patient had all three susceptible genotypes (rs7958316, rs1653624, and rs17525809), (Table 3).

**Table 3:** Association between the number of susceptible genotypesand the risk of gout.

SNPs	Contains all gout susceptibility genotypes?	Gout (n)	Hyperuricemia (n)	*P*	OR	95%CI
rs1653624 and rs7958316	Yes	86	50	0.001	2.580	1.428–4.662
	No	28	42			
rs1653624 and rs17525809	Yes	83	41	<0.001	3.971	2.174–7.254
	No	26	51			
rs7958316 and rs17525809	Yes	69	26	<0.001	4.207	2.316–7.642
	No	41	65			
rs1653624, rs7958316 and rs17525809	Yes	66	21	<0.001	5.071	2.732–9.413
	No	44	71			

### Comparison of IL-1β concentrations in the supernatants of cultured peripheral WBCs in gout and hyperuricemia patients

**Fig 2:**
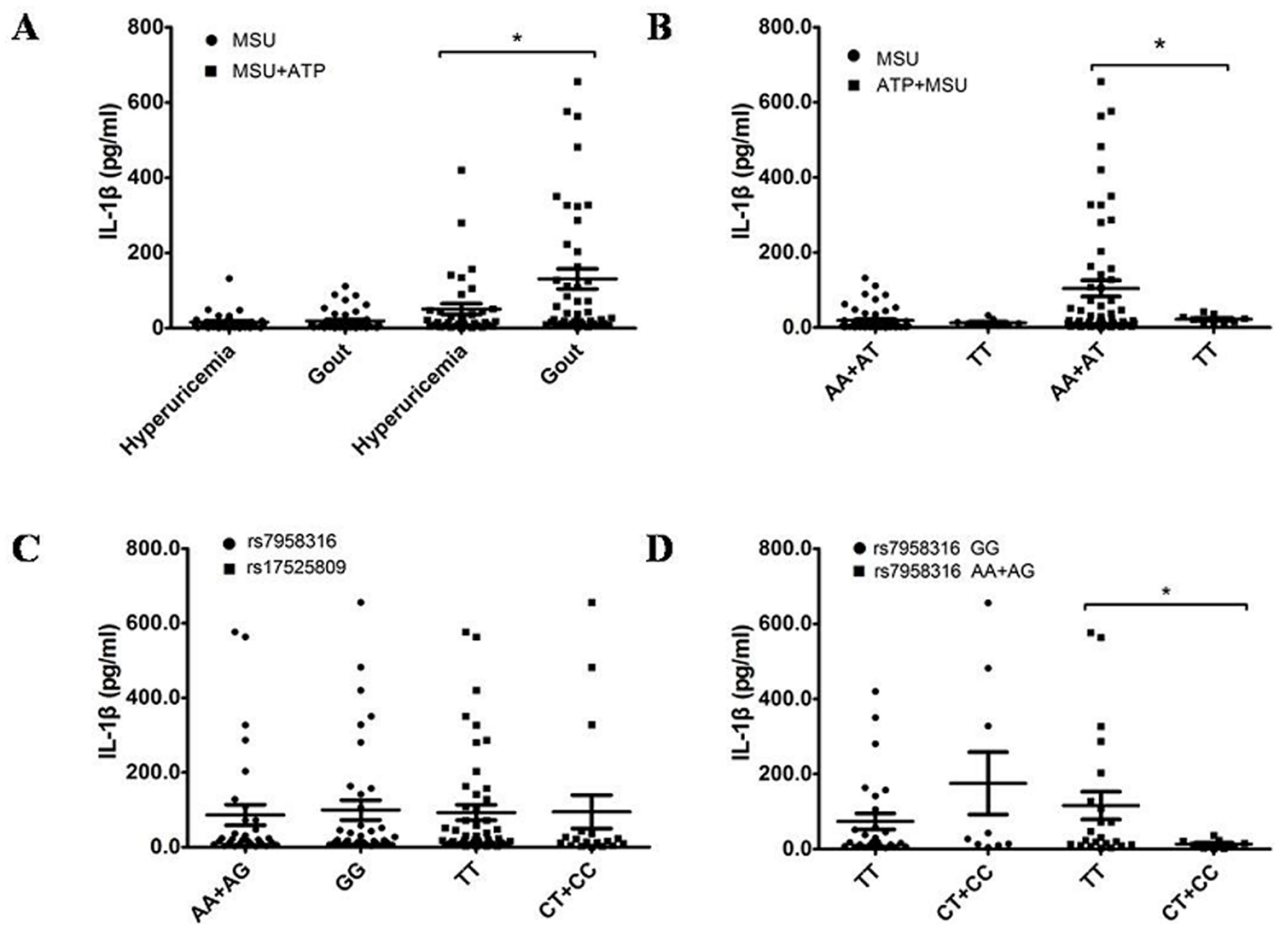
IL-1β concentrations in the supernatant of cultured peripheral leukocytes. * *P*<0.05. (**A)** There were no differences in IL-1β concentrations between the gout and hyperuricemia groups following MSU stimulation alone, but IL-1β concentrations increased significantly in gout patients following MSU + ATP stimulation. (**B)** MSU + ATP stimulation significantly elevated IL-1β in patients with rs1653624 who carried the gout susceptibility genotype AA or AT; there was no difference IL-1β concentrations after MSU stimulation alone. (**C)** There were no significant differences between patients who carried the gout susceptibility genotype of rs7958316 or rs17525809 after MSU + ATP stimulation. (**D)** IL-1β concentrations in patients who carried the rs7958316 susceptibility genotype (TT) increased significantly after MSU + ATP stimulation compared to patients who carried the rs17525809 susceptibility genotype (AA or AG), but there were no differences in patients who carried the rs17525809-non-susceptible genotypes (GG).

MSU stimulation alone produced no differences in IL-1β concentrations between the gout and hyperuricemia groups. However, gout patients produced higher concentrations of IL-1β than the hyperuricemia patients following MSU + ATP stimulation [(131.08 ± 176.11) pg/ml vs. (50.84 ± 86.10) pg/ml, *P* = 0.012)] (Fig 2A). Three SNP polymorphisms of the P2X7R ATP receptor (rs7958316, rs1653624, and rs17525809) are associated with gout pathogenesis. Therefore, IL-1β concentrations were analyzed in the supernatants of gout and hyperuricemia patients who carried all gout susceptibility genes. MSU + ATP stimulation significantly elevated IL-β levels in cell culture supernatants of patients with rs1653624 who carried the gout susceptible AA or AT genotypes [(104.20 ± 164.25) pg/ml vs. (21.90 ± 12.14) pg/ml, *P*<0.001)]. However, no difference in IL-1β concentration was observed with MSU stimulation alone (Fig 2B). No significant difference was observed between patients who carried the gout susceptible rs7958316 and rs17525809 genotypes (Fig 2C). Patients were further divided into two groups, rs7958316-susceptible genotypes (TT) and non-susceptible genotypes (CT + CC) because of the interactions between multiple SNP sites. IL-1β concentrations in samples from rs17525809 individuals with susceptible genotypes (AA or AG) were compared to non-susceptible genotypes (GG). The results demonstrated that the IL-1β concentration was significantly higher in patients with rs17525809-susceptible genotypes following MSU + ATP stimulation [(116.01 ± 173.00) pg/ml vs. (17.57 ± 14.41) pg/ml, *P* = 0.015)], but there were no differences in patients with rs7958316 non-susceptible genotypes (Fig 2D).

## Discussion

Asymptomatic hyperuricemia patients with MSU or joint tophi never develop acute gouty arthritis, but local joint inflammation may be observed. An increase in power-Doppler signals in ultrasonography, which is a surrogate for increased vascularity of inflammation, was observed in 67% of 12 asymptomatic patients with MSU deposition [32]. However, the present study found no difference in IL-1β concentrations in supernatants after MSU stimulation of cultured peripheral leukocytes between gout and hyperuricemia patients. Our results suggest that the inflammation caused by MSU crystals was not different in gout and hyperuricemia patients, and MSU crystals alone cannot induce the onset of acute gouty arthritis. However, MSU + ATP stimulation produced higher IL-1β concentrations in gout patients than those in hyperuricemia patients. These results suggest that the differences in IL-1β and other cytokine secretion caused by the ATP signaling pathway determine the onset of gout. ATP is an energy carrier that is necessary to maintain metabolism in the human body. All complex organisms undergo fluctuations in ATP levels, but changes in ATP are not the deciding factor in gout. Our results suggest that differences in P2X7R receptor function play a critical role.

The P2X7R gene is highly polymorphic. SNPs in the P2X7R coding region may affect receptor expression and function. Mutations in this receptor are divided into loss-of-function and gain-of-function [33–34]. The present study used a database to screen all of the Chinese non-synonymous coding SNP loci, and a total of eight sites were noted (Table 4). The rs1718119, rs208294, and rs17525809 loci exhibited a gain of function, and the rs7958316, rs16950860, and rs1653624 loci exhibited a loss of function. The results of the present genotyping study demonstrated that mutations in the P2X7R gene at the rs1653624, rs7958316, and rs17525809 loci were related to gout onset. Allele A of rs1653624 and rs7958316 and allele T of rs17525809 may become dangerous genes if mutated and activate gout. A hyperuricemia patient who carries these dangerous genes at these loci may be more susceptible to a gout attack.

**Table 4:** The eight selected non-synonymous coding SNPs in the P2X7R gene.

SNP ID	mRNA location (polymorphism)	MAF	Function
rs10160951	C1432G (Pro430Arg)	0.0423	NA
rs1718119	A1185G (Thr348Ala)	0.4000	Enhance IL-1β secretion [30].
rs7958316	A970G (His276Arg)	0.0124	Impair the function of P2X7R pores [35].
rs16950860	C951T (Arg270Cys)	0.0080	Weaken the function of P2X7R [29].
rs208294	C606T (His155Tyr)	0.4700	Improve affinity to ATP [36].
rs17525809	C370T (Ala76Val)	0.0499	Enhance P2X7R function, synergy with rs208294 [37].
rs1653624	A1846T (Asn568Ile)	0.0058	Hinder the normal transportation of P2X7R [38].
rs2230912	A1522G (Gln460Arg)	0.0693	Alter the structure of P2X7R [39].

Mutation at the rs1653624 gene locus converts the nucleotide sequence from AAC to ATC, which replaces asparagine (Asn) with isoleucine (Ile) at gene locus No. 568 of the P2X7R and hinders normal receptor function. This mutation is a loss-of-function mutation. Mutation at the rs7958316 gene locus converts the nucleotide sequence from CAT to CGT, which replaces histidine (His) with arginine (Arg) at gene locus No. 276 of the P2X7R. Arg is an alkaline amino acid. Previous research demonstrated that mutation at this gene locus partially damaged the P2X7R channel membrane protein function and reduced P2X7R function. This mutation is also a loss-of-function mutation. Gout risk should increase if the gout sensitivity alleles at gene loci rs1653624 and rs7958316 were A, and the loss-of-function did not occur.

Mutation at the rs17525809 gene locus converts the nucleotide sequence from GCG to GTG, which replaces alanine (Ala) with valine (Val) at gene locus No. 76. Oyanguren reported that this gene locus was closely related to multiple sclerosis. Flow cytometry revealed a greater uptake of calcium ions and ethidium bromides by P2X7R on the cell surfaces of patients with multiple sclerosis than healthy controls [40]. Mutation at this gene locus enhanced P2X7R function, and it is a gain-of-function mutation. The function of P2X7RA was enhanced when the gout-sensitive allele at gene loci of rs17525809 was mutant allele T, which rendered an individual more prone to suffer a gout attack.

MSU + ATP co-stimulated peripheral blood leukocytes in the present study, and IL-1β levels were higher in cell culture media of a patient with rs1653624 gout-sensitive genes (AA and AT) than controls. There was no difference between IL-1β levels in the cell culture media of the other two patients with gout-sensitive genes, but the IL-1β levels of a patient with two gout-sensitive genotypes increased significantly. This result suggests that the function of P2X7R was enhanced in all three of these gout-sensitive genotypes, which is consistent with previous studies. These three genotypes were also associated with an increased risk of gout in clinical settings.

The risk of developing gout in patients with hyperuricemia who carried two or more of the gout-sensitive SNP genotypes related to P2X7R function was analyzed further, and the results demonstrated that patients with two or more of these SNP genotypes were more likely to have gout. The risk of developing gout increased with increased numbers of loci. Therefore, a patient with more gout-sensitive gene loci would be more prone to suffer from gout. Result of the other gene loci, such as rs1718119, rs208294, and rs2230912, suggested no relationship with gout onset. However, the functions of different genotypes at the SNP gene loci related to the P2X7R gene are different. Any changes at SNP gene loci may alter P2X7R function. Changes in SNP gene loci exhibited limited effects on gout severity, but these changes exerted a detectable impact on gout onset. Mutation at a single SNP locus that changed only one amino acid produced receptors that maintained some of their original function. However, mutations at multiple loci that altered several amino acids affected P2X7R function. The risk of gout onset increased when P2X7R function increased to specific threshold.

Some hyperuricemia patient who did not carry the gout sensitivity gene loci discussed in this study were still prone to develop gout, which suggests that other factors affect P2X7R function. For example, changes in the sequences of non-coding and regulatory parts of the P2X7R gene or its microRNA are currently under widespread investigation. The uric acid signaling pathway also plays different roles in hyperuricemia patients. Gene polymorphisms of TLRs and the NALP3 inflammasome in the signaling pathway stimulated by MSU are related to gout onset [41], which suggests that the signaling pathway stimulated by MSU may cause more IL-1β secretion in some hyperuricemia patients and increase the risk of gout.

Gout attacks are the result of the interaction of MSU and ATP, and the SNPs present in the P2X7R gene cause dysfunction of immune cells, which may lead to the occurrence of gout. These findings provide new theoretical evidence to improve the pathogenesis of acute gouty arthritis and may explain why some hyperuricemia patients never develop acute gouty arthritis. These results may also explain the clinical phenomena of allopurinol and colchicine. Previous clinical consensus is that allopurinol induces acute gouty arthritis via the dissolution of urate crystals when used to reduce uric acid levels. However, allopurinol also significantly increases ATP levels [42], which suggests that allopurinol stimulates the P2X7R signaling pathway to induce acute gouty arthritis. Some clinicians recommend colchicine for the treatment of acute gouty arthritis, and its mechanism is related to the inhibition of leukocyte migration. Colchicine significantly inhibits gouty inflammation, but it has no effect on other inflammatory diseases. A clinical response to colchicine treatment was the basis for a gout diagnosis, but there is no plausible mechanism to explain this response. Recent scholars found that colchicine suppressed ATP-induced activation of the P2X7R signaling pathway and reduced the secretion of IL-1β [43]. This action may be the major mechanism by which colchicine prevents and treats acute gouty arthritis, and it provides indirect evidence of a role for the ATP-P2X7R signaling pathways in the pathogenesis of gout. These findings may provide a new therapeutic strategy for the prevention and treatment of gouty arthritis.

## Supporting information

S1 File Fig A) Joint involvement in rats (No. 1 rat). Fig B) The degree of joint swelling in rats. E: Normal rat joints, F: Joints in position injected with MSU was swollen. G: The swelling of foreleg ankle joint of rat No. 1 was more severe than the swelling of F. Fig C) Cytological examination of HE staining of rat joint inflammation area. K(×100), L(×400) were the results of HE staining of articular tissue in No.1 rats. M(×100), N(×400) were the results of HE staining of articular tissue of common rats. High-power microscope shows that the cells in L are mainly neutrophils of lobulated nuclei, and the cells in N are mainly lymphocytes of circular nuclei.
(DOCX)

## References

[1] Martinon F, Pétrilli V, Mayor A, Tardivel A, Tschopp J. Gout-associated uric acid crystals activate the NALP3 Inflammasome. Nature. 2006; 440(7081): 237–41. doi: 10.1038/nature04516 16407889 10.1038/nature04516

[2] Dalbeth N, Pool B, Gamble GD, Smith T, Callon KE, McQueen FM, et al Cellular characterization of the gouty tophus: a quantitative analysis. Arthritis Rheum. 2010; 62(5): 1549–56. doi: 10.1002/art.27356 20131281 10.1002/art.27356

[3] Chapman PT, Yarwood H, Harrison AA, Stocker CJ, Jamar F, Gundel RH, et al Endothelial activation in monosodium urate monohydrate crystal-induced inflammation: in vitro and in vivo studies on the roles of tumor necrosis factor alpha and interleukin-1. Arthritis Rheum. 1997; 40(5): 955–65. 9153559 10.1002/art.1780400525

[4] Dalbeth N, Haskard DO. Mechanisms of inflammation in gout. Rheumatology. 2005, 44(9): 1090–6. doi: 10.1093/rheumatology/keh640 15956094 10.1093/rheumatology/keh640

[5] Martinon F. Mechanisms of uric acid crystal-mediated autoinflammation. Immunol Rev. 2010; 233(1): 218–32. doi: 10.1111/j.0105-2896.2009.00860.x 20193002 10.1111/j.0105-2896.2009.00860.x

[6] Liu-Bryan R, Scott P, Sydlaske A, Rose DM, Terkeltaub R. Innate immunity conferred by Toll-like receptors 2 and 4 and myeloid differentiation factor 88 expression is pivotal to monosodium urate monohydrate crystal-induced inflammation. Arthritis Rheum. 2005; 52(9): 2936–46. doi: 10.1002/art.21238 16142712 10.1002/art.21238

[7] Church LD, Cook GP, McDermott MF. Primer: inflammasomes and interleukin 1β in inflammatory disorders. Nat Clin Pract Rheumatol. 2008; 4(1): 34–42. doi: 10.1038/ncprheum0681 18172447 10.1038/ncprheum0681

[8] Campion EW, Glynn RJ, DeLabry LO. Asymptomatic hyperuricemia. Risks and consequences in the Normative Aging Study. Am J Med. 1987; 82(3): 421–6. 3826098 10.1016/0002-9343(87)90441-4

[9] Hall AP, Barry PE, Dawber TR, McNamara PM. Epidemiology of gout and hyperuricemia: a long-term population study. Am J Med. 1967; 42(1): 27–37. 6016478 10.1016/0002-9343(67)90004-6

[10] Roubenoff R. Gout and hyperuricemia. Rheum Dis Clin North Am. 1990; 16(3): 539–50. 2217957

[11] Rouault T, Caldwell DS, Holmes EW. Aspiration of the asymptomatic metatarsophalangeal joint in gout patients and hyperuricemic controls. Arthritis Rheum. 1982; 25(2): 209–12. 7066051 10.1002/art.1780250215

[12] Pascual E, Jovaní V. A quantitative study of the phagocytosis of urate crystals in the synovial fluid of asymptomatic joints of patients with gout. Br J Rheumatol. 1995; 34(8): 724–6. 7551655 10.1093/rheumatology/34.8.724

[13] Howard RG, Pillinger MH, Gyftopoulos S, Thiele RG, Swearingen CJ, Samuels J. Reproducibility of musculoskeletal ultrasound for determining monosodium urate deposition: concordance between readers. Arthritis Care Res. 2011; 63(10): 1456–62. 10.1002/acr.20527 PMC3183112 21702086

[14] Pineda C, Amezcua-Guerra LM, Solano C, Rodriguez-Henríquez P, Hernández-Díaz C, Vargas A. Joint and tendon subclinical involvement suggestive of gouty arthritis in asymptomatic hyperuricemia: an ultrasound controlled study. Arthritis Res Ther. 2011; 13(1):R4 doi: 10.1186/ar3223 21241475 10.1186/ar3223 PMC3241349

[15] De Miguel E, Puig JG, Castillo C, Peiteado D, Torres RJ, Martín-Mola E. Diagnosis of gout in patients with asymptomatic hyperuricaemia: a pilot ultrasound study. Ann Rheum Dis. 2012; 71(1): 157–8. doi: 10.1136/ard.2011.154997 21953340 10.1136/ard.2011.154997

[16] Dalbeth N, House ME, Aati O, Tan P, Franklin C, Horne A. Urate crystal deposition in asymptomatic hyperuricaemia and symptomatic gout: a dual energy CT study. Ann Rheum Dis. 2015; 74(5): 908–11. doi: 10.1136/annrheumdis-2014-206397 25637002 10.1136/annrheumdis-2014-206397

[17] Sun Y, Ma L, Zhou Y, Chen H, Ding Y, Zhou J, et al Features of urate deposition in patients with gouty arthritis of the foot using dual-energy computed tomography. Int J Rheum Dis. 2015; 18(5): 560–7. doi: 10.1111/1756-185X.12194 24238356 10.1111/1756-185X.12194

[18] Puig JG, De Miguel E, Castillo MC, Rocha AL, Martínez MA, Torres RJ. Asymptomatic hyperuricemia: impact of ultrasonography. Nucleosides Nucleotides Nucleic Acids. 2008; 27(6):592–5. doi: 10.1080/15257770802136040 18600510 10.1080/15257770802136040

[19] López Redondo MJ, Requena L, Macía M, Schoendorff C, Sánchez Yus E, Robledo A. Fingertip tophi without gouty arthritis. Dermatology. 1993; 187(2): 140–3. 8358105 10.1159/000247226

[20] Shmerling RH, Stern SH, Gravallese EM, Kantrowitz FG. Tophaceous deposition in the finger pads without gouty arthritis. Arch Intern Med. 1988; 148(8): 1830–2. 3401106

[21] Hollingworth P, Scott JT, Burry HC. Nonarticular gout: hyperuricemia and tophus formation without gouty arthritis. Arthritis Rheum. 1983; 26(1): 98–101. 6824510 10.1002/art.1780260117

[22] Hillman KA, Burnstock G, Unwin RJ. The P2X7 ATP receptor in the kidney: a matter of life or death? Nephron Exp Nephrol. 2005; 101(1): e24–e30. doi: 10.1159/000086036 15925905 10.1159/000086036

[23] Tao JH, Zhang Y, Li XP. P2X7R: A potential key regulator of acute gouty arthritis. Semin Arthritis Rheum. 2013; 43(3): 376–80. doi: 10.1016/j.semarthrit.2013.04.007 23786870 10.1016/j.semarthrit.2013.04.007

[24] Buell GN, Talabot F, Gos A, Lorenz J, Lai E, Morris MA, et al Gene structure and chromosomal localization of the human P2X7 receptor. Recept Channels. 1998; 5(6): 347–54. 9826911

[25] Sorge RE, Trang T, Dorfman R, Smith SB, Beggs S, Ritchie J, et al Genetically determined P2X7 receptor pore formation regulates variability in chronic pain sensitivity. Nat Med. 2012; 18(4): 595–9. doi: 10.1038/nm.2710 22447075 10.1038/nm.2710 PMC3350463

[26] Jindrichova M, Bhattacharya A, Rupert M, Skopek P, Obsil T, Zemkova H. Functional characterization of mutants in the transmembrane domains of the rat P2X7 receptor that regulate pore conductivity and agonist sensitivity. J Neurochem. 2015; 133(6): 815–27. doi: 10.1111/jnc.13078 25712548 10.1111/jnc.13078

[27] Shemon AN, Sluyter R, Fernando SL, Clarke AL, Dao-Ung LP, Skarratt KK, et al A Thr-357 to Ser polymorphism in homozygous and compound heterozygous subjects causes absent or reduced P2X7 function and impairs ATP-induced mycobacterial killing by macrophages. J Biol Chem. 2006; 281(4): 2079–86. doi: 10.1074/jbc.M507816200 16263709 10.1074/jbc.M507816200

[28] Sluyter R, Shemon AN, Wiley JS. Glu496 to Ala polymorphism in the P2X7 receptor impairs ATP-induced IL-1 beta release from human monocytes. J Immunol. 2004; 172(6): 3399–405. 15004138 10.4049/jimmunol.172.6.3399

[29] Wesselius A, Bours M J, Arts I C, Theunisz EH, Geusens P, Dagnelie PC. The P2X(7) loss-of-function Glu496Ala polymorphism affects ex vivo cytokine release and protects against the cytotoxic effects of high ATP-levels. BMC Immunol. 2012; 13: 64 doi: 10.1186/1471-2172-13-64 23210974 10.1186/1471-2172-13-64 PMC3526505

[30] Stokes L, Fuller SJ, Sluyter R, Skarratt KK, Gu BJ, Wiley JS. Two haplotypes of the P2X(7) receptor containing the Ala-348 to Thr polymorphism exhibit a gain-of-function effect and enhanced interleukin-1beta secretion. FASEB J. 2010; 24(8): 2916–27. doi: 10.1096/fj.09-150862 20360457 10.1096/fj.09-150862

[31] Campion EW, Glynn RJ, DeLabry LO. Asymptomatic hyperuricemia. Risks and consequences in the Normative Aging Study. Am J Med. 1987; 82(3): 421–6. 3826098 10.1016/0002-9343(87)90441-4

[32] Puig JG, De Miguel E, Castillo MC, Rocha AL, Martínez MA, Torres RJ. Asymptomatic hyperuricemia: impact of ultrasonography. Nucleosides Nucleotides Nucleic Acids. 2008; 27(6): 592–5. doi: 10.1080/15257770802136040 18600510 10.1080/15257770802136040

[33] Jiang LH, Baldwin JM, Roger S, Baldwin S. Insights into the molecular mechanisms underlying mammalian P2X7 receptor functions and contributions in diseases, revealed by structural modeling and single nucleotide polymorphisms. Front Pharmacol. 2013; 4: 55 doi: 10.3389/fphar.2013.00055 23675347 10.3389/fphar.2013.00055 PMC3646254

[34] Ursu D, Ebert P, Langron E, Ruble C, Munsie L, Zou W, et al Gain and loss of function of P2X7 receptors: mechanisms, pharmacology and relevance to diabetic neuropathic pain. Mol Pain. 2014; 10:37 doi: 10.1186/1744-8069-10-37 24934217 10.1186/1744-8069-10-37 PMC4072620

[35] Solini A, Simeon V, Derosa L, Orlandi P, Rossi C, Fontana A, et al Genetic interaction of P2X7 receptor and VEGFR-2 polymorphisms identifies a favorable prognostic profile in prostate cancer patients. Oncotarget. 2015; 6(30): 28743 doi: 10.18632/oncotarget.4926 26337470 10.18632/oncotarget.4926 PMC4745689

[36] Cabrini G, Falzoni S, Forchap SL, Pellegatti P, Balboni A, Agostini P, et al A His-155 to Tyr polymorphism confers gain-of-function to the human P2X7 receptor of human leukemic lymphocytes. The Journal of Immunology. 2005; 175(1): 82–9. 15972634 10.4049/jimmunol.175.1.82

[37] Bhattacharya A, Biber K. The microglial ATP-gated ion channel P2X7 as a CNS drug target. Glia. 2016; 64(10): 1772–87. doi: 10.1002/glia.23001 27219534 10.1002/glia.23001

[38] Wiley JS, Dao-Ung LP, Li C, Shemon AN, Gu BJ, Smart ML, et al An Ile-568 to Asn polymorphism prevents normal trafficking and function of the human P2X7 receptor. J Biol Chem. 2003; 278(19): 17108–13. doi: 10.1074/jbc.M212759200 12586825 10.1074/jbc.M212759200

[39] Roger S, Mei ZZ, Baldwin JM, Dong L, Bradley H, Baldwin SA, et al Single nucleotide polymorphisms that were identified in affective mood disorders affect ATP-activated P2X7 receptor functions. J Psychiatr Res. 2010; 44(6): 347–55. doi: 10.1016/j.jpsychires.2009.10.005 19931869 10.1016/j.jpsychires.2009.10.005

[40] Oyanguren-Desez O, Rodríguez-Antigüedad A, Villoslada P, Domercq M, Alberdi E, Matute C. Gain-of-function of P2X7 receptor gene variants in multiple sclerosis. Cell Calcium. 2011; 50(5): 468–72. doi: 10.1016/j.ceca.2011.08.002 21906809 10.1016/j.ceca.2011.08.002

[41] Qing YF, Zhou JG, Zhang QB, Wang DS, Li M, Yang QB, et al Association of TLR4 Gene rs2149356 polymorphism with primary gouty arthritis in a case-control study. PLOS ONE. 2013; 8(5):e64845 doi: 10.1371/journal.pone.0064845 23738004 10.1371/journal.pone.0064845 PMC3667827

[42] Hirsch GA, Bottomley PA, Gerstenblith G, Weiss RG. Allopurinol acutely increases adenosine triphospate energy delivery in failing human hearts. J Am Coll Cardiol. 2012; 59(9): 802–8. doi: 10.1016/j.jacc.2011.10.895 22361399 10.1016/j.jacc.2011.10.895 PMC4208309

[43] Marques-da-Silva C, Chaves MM, Castro NG, Coutinho-Silva R, Guimaraes MZ. Colchicine inhibits cationic dye uptake induced by ATP in P2X2 and P2X7 receptor-expressing cells: implications for its therapeutic action. Br J Pharmacol. 2011; 163(5): 912–26. doi: 10.1111/j.1476-5381.2011.01254.x 21306580 10.1111/j.1476-5381.2011.01254.x PMC3130939

