# Supplementary material for: Single nucleotide polymorphisms associated with P2X7R function regulate the onset of gouty arthritis
Source: PLoS One. 2017 Aug 10;12(8):e0181685. doi: 10.1371/journal.pone.0181685 (PMC5552250; doi:10.1371/journal.pone.0181685)
Supplement: S1 File — Fig A) Joint involvement in rats (No. 1 rat). Fig B) The degree of joint swelling in rats. E: Normal rat joints, F: Joints in position injected with MSU was swollen. G: The swelling of foreleg ankle joint of rat No. 1 was more severe than the swelling of F. Fig C) Cytological examination of HE staining of rat joint inflammation area. K(×100), L(×400) were the results of HE staining of articular tissue in No.1 rats. M(×100), N(×400) were the results of HE staining of articular tissue of common rats. High-power microscope shows that the cells in L are mainly neutrophils of lobulated nuclei, and the cells in N are mainly lymphocytes of circular nuclei. (DOCX) [file pone.0181685.s001.docx]

**ATP and MSU synergistically induce gout flare in rats**

Recently, our group has established an animal model of gout based on the new pathogenesis of gouty arthritis induced by MSU together with ATP. By adopting the Coderre method as well as intravenous injecting ATP, we aimed to inspect the severity and symptoms of arthritis in animal models. In this study, 20 SD male rats have been selected and injected with MSU into their ankle joints. At the same time, 10 rats have been selected randomly and injected with different doses of ATP through tail vein. No significant differences were observed in joint swelling degrees between the rats with and without ATP tail vein injection. Of note, one rat with ATP injection showed different swelling symptoms in ankles from any other rats. And we have labeled this one as No. 1 rat. The main differences include:

(1) The affected joints were different: swelling generally occurred in joints with MSU injection, while it has attacked some other parts of the No. 1 rat, and the joints with MSU injection turn out to be less swollen (Fig A in S1 File).

(2) The degree and developing speed of joint swelling: severe swelling occurred to the No. 1 Rat, with obvious symptoms, including hot redness of the skin (Fig B in S1 File). The symptoms of No. 1 rat rapidly deteriorated and reached a peak within 24 hours after the injection of MSU and ATP, while peak periods in other rats were 3 days after injection of MSU.

(3) Distinct histopathologic characteristic: Using HE staining of joint tissues, we sought to clarify cell types infiltrated in rat joint according to the karyomorphism under High-power microscope. It was clearly shown that joint tissues in rat No. 1 were dominant neutrophilic infiltrated, while others were mainly with high lymphocytic infiltration (Fig C in S1 File).


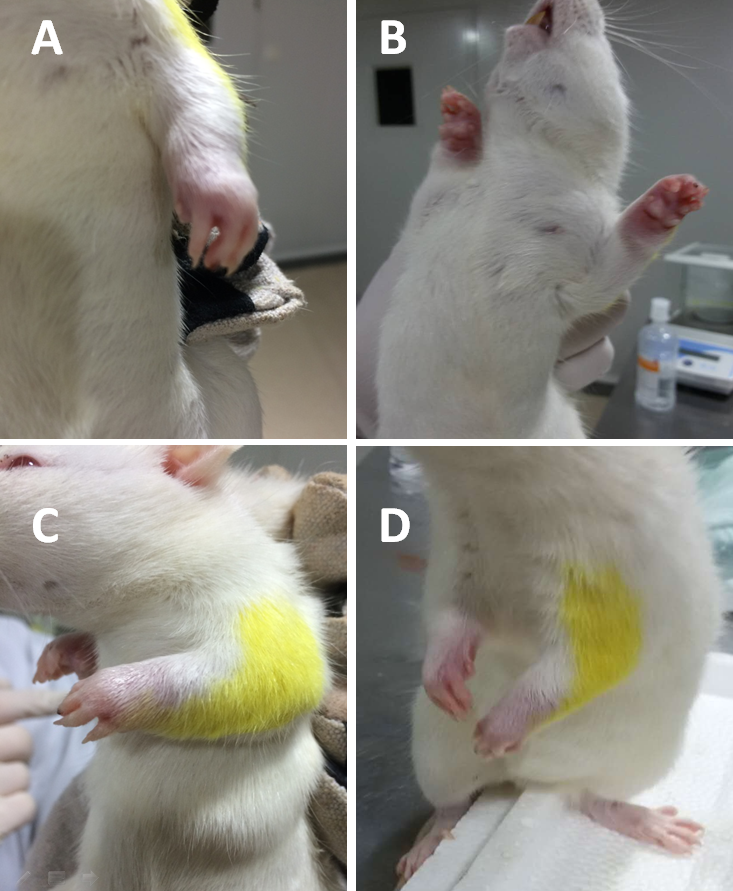


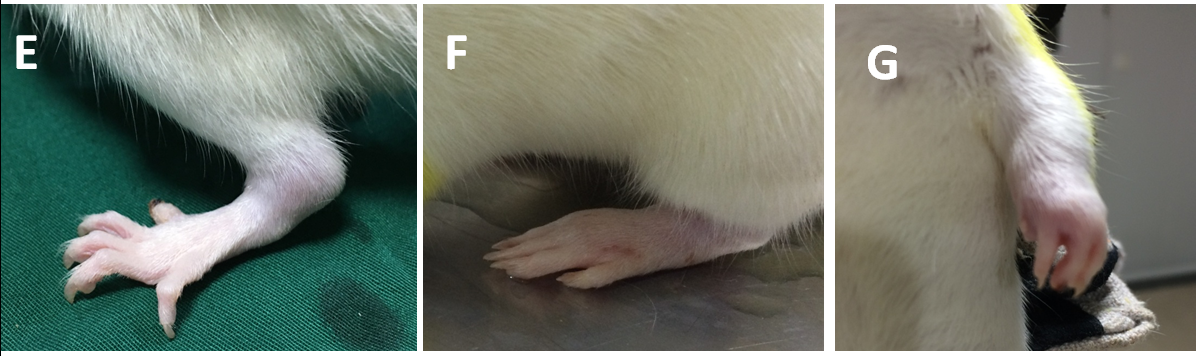


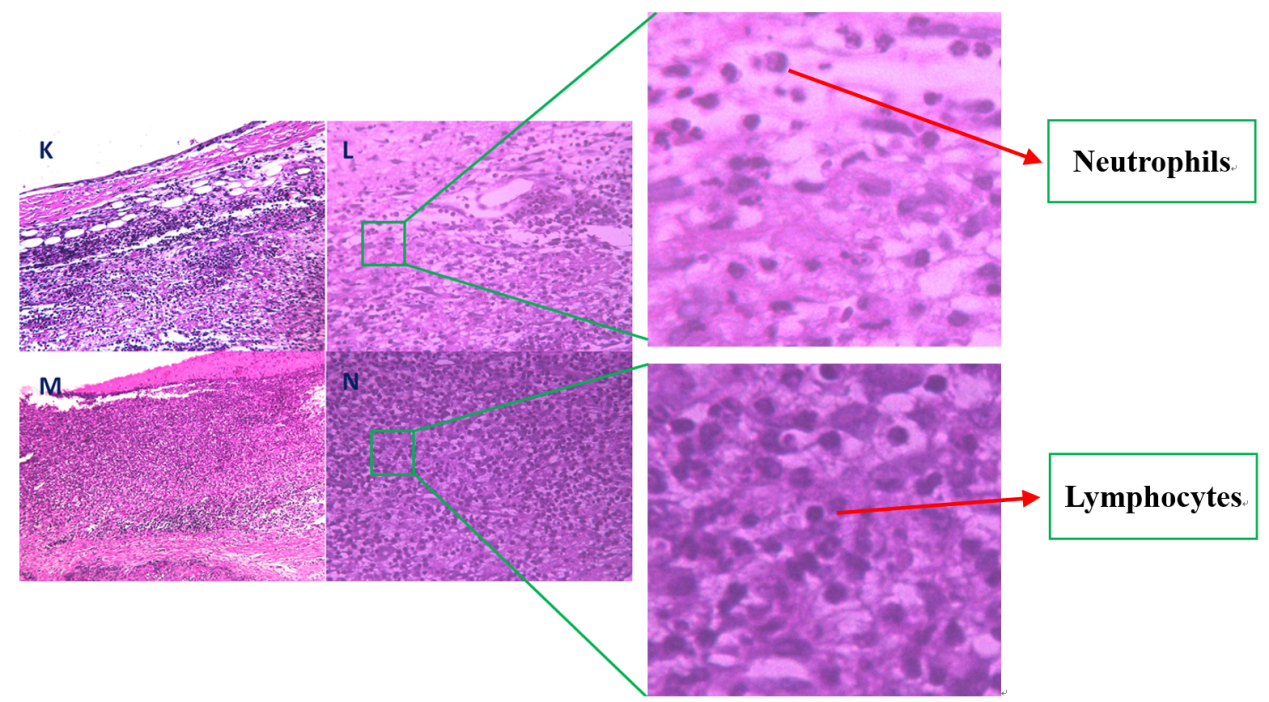


S1 File. Fig A) Joint involvement in rats (No. 1 rat). Fig B) The degree of joint swelling in rats. E: Normal rat joints, F: Joints in position injected with MSU was swollen. G: The swelling of foreleg ankle joint of rat No. 1 was more severe than the swelling of F. Fig C) Cytological examination of HE staining of rat joint inflammation area. K（×100）、L（×400）were the results of HE staining of articular tissue in No.1 rats. M（×100）、N（×400）were the results of HE staining of articular tissue of common rats. High-power microscope shows that the cells in L are mainly neutrophils of lobulated nuclei, and the cells in N are mainly lymphocytes of circular nuclei.

We’ve found that the No. 1 rat showed more similarities with human in the symptoms of gout flare, and the symptoms of other rats are more similar with non-specific inflammations stimulated by foreign matters, based on the analysis of symptoms and the pathological manifestations of tissues. Therefore, previous models for gouty arthritis merely induced by MSU might be non-specific inflammations, rather than gout flares. Only the models of gouty arthritis of the pathogenesis induced by both ATP and MSU may be regarded as real gout flares. Gout flare is influenced by many factors, including genetics, drug delivery methods and doses of ATP and MSU. Though only one rat with “real” gout attack has been observed in this research, it’s likely that ATP plays a role in the pathogenesis of gout flare.
